# Short Practical Notes for Medical Motorists

**Published:** 1909-06-26

**Authors:** 


### SHORT PRACTICAL NOTES FOR MEDICAL MOTORISTS.

It frequently happens that the branches of split pins, not bent back upon a bolt or nut, lacerate the driver's hands when they come in contact with them. The threatening branches should be turned back with the hand and then flattened down with a copper hammer.

After bringing a car to a stop the change-gear lever should always be brought back to its neutral position. This removes the possibility of the driver, as he attempts to start the engine, suddenly pulling the car on to him. This mishap will probably administer a nasty knock, if, indeed, it is not attended by a much more serious injury.

Unless the driver of a motor-car is certain that there is no vehicle close behind him he should never make a sudden reduction of speed or come to a quick stop without giving a warning signal. The customary way of announcing the intention of slowing or stopping is to raise one hand in the air, so that those following may see it readily. Rear-end collisions are undesirable, to say the least, and there are many occasions when they may easily occur if this simple method of signalling is neglected.

Few motorists except those of long experience realise the strain that is put upon the steering mechanism by twisting the wheels around by means of the steering-wheel when the car is stationary. Whenever this is done an unnecessary amount of wear is put upon the steering mechanism, and sometimes even the steering connections may be bent.

Testing a sparking plug on the outside of the cylinder, with no compression to influence it, is not a correct exposition of its efficiency in producing desired results within the cylinder. The plug may spark beautifully when under observation, but when returned to its proper position no perfect result may be obtained. Faulty ignition, if everything connected with the contact-maker, battery, and coil is in order, is usually due to the points of the plug being too far apart. The points should be placed as close together as possible without being liable to fouling by small particles of carbon. Closeness of points not only favours production of sparks under compression, but it interposes less resistance to the current passing across the gap, and thus makes it less liable to follow some other track. "Viator."

